# A New Controlled-Release Material Containing Metronidazole and Doxycycline for the Treatment of Periodontal and Peri-Implant Diseases: Formulation and In Vitro Testing

**DOI:** 10.1155/2019/9374607

**Published:** 2019-03-05

**Authors:** Livia Nastri, Alfredo De Rosa, Vincenza De Gregorio, Vincenzo Grassia, Giovanna Donnarumma

**Affiliations:** ^1^Multidisciplinary Department of Medical-Surgical and Odontostomatological Specialties, University of Campania “Luigi Vanvitelli”, 80100 Naples, Italy; ^2^Center for Advanced Biomaterials for HealthCare@CRIB, Istituto Italiano di Tecnologia, Largo Barsanti e Matteucci 53, 80125 Naples, Italy; ^3^Department of Experimental Medicine, Section of Microbiology and Clinical Microbiology, University of Campania “Luigi Vanvitelli”, 80100 Naples, Italy

## Abstract

**Background:**

Several locally administered antimicrobials have been studied in the literature as adjunctive or primary treatments for periodontitis and peri-implantitis with conflicting results.

**Objective:**

The aim of this study was twofold: (1) the formulation of a controlled-release material containing metronidazole and doxycycline; (2) an *in vitro* evaluation of its antibacterial properties against planktonic and biofilm species involved in periodontal and peri-implant diseases.

**Methods:**

Doxycycline (10 mg/ml) and metronidazole (20 mg/ml) were incorporated into a hydroxyethylcellulose-polyvinylpyrrolidone-calcium polycarbophil gel. Three milliliters of gel were dialyzed against Dulbecco's phosphate-buffered saline for 13 days. Antibiotics release at 3, 7, 10, and 13 days was determined spectroscopically. The inhibitory activity of the experimental gel was tested against *A. actinomycetemcomitans*, *S. sanguinis*, *P. micra*, and *E. corrodens* with an agar diffusion test, an inactivation biofilm test, and a confocal laser scanning microscope study (CLSMS) for *S. sanguinis* up to 20 days.

**Results:**

After 13 days, the released doxycycline was 9.7% (at 3 days = 1.2 mg; 7 days = 0.67 mg; 10 days = 0.76 mg; 13 days = 0.29 mg), while metronidazole was 67% (30 mg, 6.8 mg, 2.5 mg, and 0.9 mg at the same intervals). The agar diffusion test highlights that the formulated gel was active against tested microorganisms up to 312 h. Quantitative analysis of biofilm formation for all strains and CLSMS for *S. sanguinis* showed a high growth reduction up to 13 days.

**Conclusions:**

The *in vitro* efficacy of the newly formulated gel was confirmed both on planktonic species and on bacterial biofilm over a period of 13 days. The controlled-release gel containing metronidazole and doxycycline had an optimal final viscosity and mucoadhesive properties. It can be argued that its employment could be useful for the treatment of periodontal and peri-implant diseases, where conventional therapy seems not successful.

## 1. Introduction

Periodontal diseases are a group of inflammatory conditions affecting the supportive structures of the teeth characterized by destruction of the periodontal ligament, resorption of the alveolar bone, and migration of the junctional epithelium along the root surface with the resultant formation of a pocket. The periodontal pocket provides an ideal environment for the growth and proliferation of potentially pathogenic microorganisms.

Current options for periodontal therapy include the removal of the bacterial deposits from the tooth surface, shifting the microorganism biofilms toward a periodontally healthy and less virulent composition and modulating the host response. While several treatment approaches are therefore available to the clinician, conventional scaling and root planing (SRP), in conjunction with proper plaque control, remains the primary treatment option for the clinician [1]. The efficacy of SRP as a part of the nonsurgical treatment of chronic periodontitis has been established with a general consistency of results, based on the improvement of measurable endpoints that include clinical attachment level, probing depth, bleeding on probing, and an alteration in the subgingival microflora [2–7]. Although it is generally concluded that nonsurgically performed pocket/root debridement is an effective treatment approach, it is also evident from the literature that various patient- and site-related factors may influence the healing response to treatment or its long-term stability [2–4]. In addition, SRP in moderately deep pockets may be technically demanding, time-consuming [8], lead to incomplete debridement [9], and not eradicate species that can penetrate epithelial cells and subepithelial connective tissue of the periodontium [10]. An environment similar to periodontal pocket, with comparable even if more severe morphological and immunohistochemical features of the inflammatory infiltrate, is created by the destructive process around dental implants known as peri-implantitis [11, 12]. In peri-implantitis, the presence of a nonsmoothable surface and of the implant treads impairs the ability to cleanse the surface and may hinder effective nonsurgical debridement of the infected implant, decreasing the healing potential [13].

Therefore, antibiotics and antiseptics have also been used to treat moderate to severe periodontal disease [14, 15] as peri-implantitis [16, 17]. However, the high doses of systemic antibiotics required to achieve therapeutic concentrations at target sites led to an increased awareness of side-effects, including allergies, gastrointestinal disorders, and the development of antibiotic resistance [18, 19].

Over the last 25 years, locally delivered, anti-infective pharmacological agents have been employed in attempt to treat local bacterial infections associated with gingivitis and periodontitis [8, 20, 21].

The rationale for a locally delivered antibacterial therapy is that the local route of drug delivery can accomplish, compared with systemic routes of administration, up to 100-fold higher drug therapeutic doses in the subgingival areas [22, 23]. However, in order to achieve a positive effect on periodontal parameters, local application must fulfill 3 criteria: (1) reach the intended site of action; (2) achieve therapeutic concentration; and (3) last for a sufficient amount of time [8, 24]. Moreover, the environment of the periodontal pocket poses two great challenges to local delivery agents: (1) the bacteria are organized in biofilm with an increased resistance compared to planktonic forms [25] and (2) the presence of the gingival crevicular fluid (GCF), which greatly increases the clearance of the drug in the pocket. Experimental evidence suggests that currently available local delivery drugs are indeed not able to satisfy the abovementioned criteria, in some cases for lack of substantivity or for lack of sufficient antibacterial power to counteract the formation of the biofilm [8, 24, 26]. Several antibiotics have been used in periodontal treatments [21].

Doxycycline is a well-known broad-spectrum antibiotic, with antimicrobial activity against the subgingival microflora. Among its properties, a remarkable feature is the ability to bind to dentin surface and be substantive, maintaining bacteriostatic concentration useful against most of the periodontopathogens [27]. *In vitro* testing has shown that *Porphyromonas gingivalis*, *Prevotella intermedia*, *Campylobacter rectus*, and *Fusobacterium nucleatum* are susceptible to doxycycline at concentrations of 6.0 *μ*g/ml [28]. Other suspected periodontal pathogens have a susceptibility to doxycycline ranging from 0.1 to 2.0 *μ*g/ml, while in biofilms, the necessary minimum inhibitory concentrations (MICs) are at least 50 times higher [29–33]. In addition to its antibacterial activity, the inhibitory effects of doxycycline especially against polymorphonuclear neutrophil-derived and bacterial-derived collagenases (matrix metalloproteinases, MMP) have been reported, [34, 35].

Metronidazole was introduced in the 1960s for treatment of vaginal trichomoniasis [36, 37]. Lately, it has been employed on acute necrotic ulcerative gingivitis (ANUG). Metronidazole has been used in the treatment of periodontal diseases because it is accumulated by obligated anaerobic bacteria [18, 38]. Metronidazole showed to be effective against *Aggregatibacter actinomycetemcomitans*, *Prevotella intermedia*, *Porphyromonas gingivalis*, *Tannerella forsythensis*, *Prevotella nigrescens* [39, 40], *Streptococcus sanguinis*, *Parvimonas micra*, and *Eikenella corrodens* [41–44]. Different delivering systems of metronidazole have been proposed and investigated with some positive results [20, 24, 45–47].

Tetracyclines and metronidazole were reported to be effective for the treatment of periodontal diseases [48, 49].

Doxycycline hydrochloride was chosen for this study for its activity against putative periodontal pathogens and its ability to inhibit collagenases [50, 51].

A combination of metronidazole and doxycycline has been largely employed in papulopustular rosacea [52] and pelvic inflammatory diseases [53]. Doxycycline was both employed at antibacterial doses and subantimicrobial doses. In endodontics, a local antimicrobial paste that combines metronidazole and minocycline (where minocycline is comparable to doxycycline in antibacterial properties) is being largely used to “sterilize” root canals of necrotic teeth [54, 55].

To date, to the best of our knowledge, there is no commercially available product that couples the release of doxycycline and metronidazole for a sustained period of time.

Therefore, the objective of this study was twofold: (1) the formulation of a controlled-release material containing metronidazole and doxycycline (MET/DOX Gel); (2) an *in vitro* evaluation of its antibacterial properties against planktonic and biofilm species involved in periodontal and peri-implant infections.

## 2. Materials and Methods

This study was conducted in the laboratories of the Department of Experimental Medicine, University of Campania “Luigi Vanvitelli,” Naples, Italy.

The study was broadly divided into two phases:

- *Pharmacological phase*: formulation of a controlled-release material able to incorporate and release the selected active compounds; *in vitro* testing to evaluate drug release timing and modality
- *Microbiological phase*: *in vitro* test to evaluate the antimicrobial activity of the controlled-release material against planktonic and biofilm species

### 2.1. Pharmacological Phase

#### 2.1.1. Mucoadhesive Formulation Preparation

**Figure 1:**
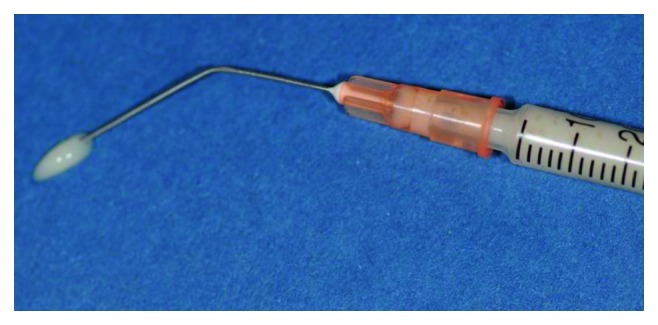
Light yellow, clear, and syringeable formulation. MET/DOX gel was flowable and mucoadhesive.

Different mucoadhesive polymers and proportions of the compounds were used in a preliminary test. The prepared gels were evaluated for their *in vitro* drug release and rheological behaviour (data not shown). The gel with appropriate balance of the above-examined parameters was selected for microbiological evaluation (Figure 1).

The tested formulation was prepared by dissolving the hydroxyethylcellulose (Natrosol 250-HHX-Pharm Merck, Darmstadt, Germany), polyvinylpyrrolidone (Polyvidon 25 Merck, Darmstadt, Germany), and polycarbophil calcium (Beta Pharma Inc., New Haven, USA) in a solution of Dulbecco's phosphate-buffered saline (DPBS, 0.0095 M, pH 7.2) (Lonza, Milano, Italy) under mechanical agitation (10 min. at 200 rpm), so that the final solution contained 3% (w/w) hydroxyethylcellulose, 2% (w/w) polyvinylpyrrolidone, and 1% (w/w) calcium polycarbophil. Once the matrix gel was formed, doxycycline-hyclate (Doxy-h) (Calbiochem, Darmstadt, Germany) and metronidazole (MET) (Farmalabor, Bari, Italy) were added at a concentration of 10 mg/ml and 20 mg/ml, respectively, stirring until homogeneity was reached. The gel thus obtained was stored at 4°C.

#### 2.1.2. In Vitro Test for Evaluating the Timing of Drug Release

For the evaluation of the timing and modality of release of the antibiotics, a solution of 3 ml of gel containing the 2 drugs (20 mg/ml and 10 mg/ml, respectively) in the proportions described above was prepared. The gel was introduced into a dialysis tube, equipped with dialysis membranes (11.5 mm diameter, cut off 1 kDa), (Spectra/Por, Rancho Dominquez, USA) and dialyzed against 40 ml of DPBS buffer (pH 7.2) under constant agitation (100 rpm) at 37°C. Dialysis lasted for 13 days with change of external medium on the 3^rd^, 7^th^, 10^th^, and 13^th^ day.

The release of the two antibiotics in the dialysis medium was determined by a spectrophotometer (UV-VIS DU640 Beckman, Palo Alto, CA, USA) by reading the absorbance (previously experimentally determined at 272 nm and 320 nm for Doxy-h and MET, respectively); measurements were performed in duplicate during the experiment against a blank consisting of a dialyzed gel without the addition of the antibiotics (placebo). The quantitative determination of the released antibiotics was obtained using a calibration curve that relates the absorbance with the concentration of the antibiotic (*r* > 0.9 over the concentration range 10–2000 *μ*g ml^−1^). The *r*-value was based on test-retest reliability and indicates the repeatability of test scores with the passage of time, while the standard error of measurement was lower of 1%.

To avoid changes in volume, after spectrophotometric reading, the sample was again introduced into the vessel of dialysis. Each test was repeated in triplicate.

#### 2.1.3. Rheological Measurements

Rheological measurements were performed using an oscillatory rheometer (Physica MCR301, Anton Paar, Germany) equipped with a parallel plate geometry (plate diameter of 25 mm, gap 0.5 mm) and a Peltier temperature control. In particular, flow curves and viscosity curves (shear stress and dynamic viscosity vs shear rate, respectively) were determined at 25°C over shear rates ranging from 0.1 to 400°s^−1^. Measurements were run in triplicate.

### 2.2. Microbiological Phase

#### 2.2.1. Bacterial Strains and Culture Conditions

*Aggregatibacter actinomycetemcomitans* (ATCC3718), *Streptococcus sanguinis (*ATCC10556), *Parvimonas micra (*ATCC33270), and *Eikenella corrodens (*ATCC23834) were selected (LGC Standards SRL, Milan, Italy). All bacterial strains were routinely grown in brain heart infusion broth (BHI) (Oxoid, Milan, Italy) or on BHI agar plates (Oxoid, Milan, Italy) and incubated for 24 hours at 37°C in jars (BBL GasPak System, Becton Dickinson & Co., Cockeysville, MD, USA) in air supplemented with 5% CO_2_, 90% N_2_, and 5% H_2_ (BBL GasPak CO2 system envelopes, Becton Dickinson & Co., Cockeysville, MD, USA) for *S. sanguinis, P. micra,* and *E. corrodens* or in microaerophilic atmosphere with 4–10% CO_2_ (BBL CampyPak, Becton Dickinson & Co., Cockeysville, MD, USA) for *A. actinomycetemcomitans.*

#### 2.2.2. Agar Diffusion Test of the MET/DOX Gel

Subgingival bacteria were grown on selective medium and incubated under standard conditions. For each bacterial strain, five colonies were collected, inoculated into BHI, and incubated at 37°C for 18 hours in microaerophilic atmosphere (*A. actinomycetemcomitans)* or under anaerobic conditions (*S. sanguinis*, *P. micra*, and *E. corrodens)*.

Bacterial density was related to McFarland standards (McFarland 1 = 3 × 10^8^ CFU ml^−1^) (BioMérieux, Florence, Italy). Briefly, 1 ml of bacterial suspension, with a turbidity equivalent to 1.0 McFarland standard, was used to evenly inoculate freshly prepared agar chocolate plates (Oxoid, Milan, Italy). The excessive suspension was removed with a pipette, and the plates were dried for 30 min at room conditions. Successively, a blank disk of 13 mm diameter (Becton Dickinson & Co., Cockeysville, MD, USA) was placed in the middle of the agar plate and coated with placebo or MET/DOX gel (20 mg/ml and 10 mg/ml, respectively). The agar plates were then incubated at 37°C in anaerobic or microaerophilic conditions for 24, 48, 72, 96, 120, 144, 168, 192, 240, and 312 h. To test the antibacterial activity of the MET/DOX gel over prolonged periods of time, each disk (not recharged with gel) was transferred daily in a new chocolate agar plate inoculated as described above. The diameter of bacterial growth inhibition zones was measured with a millimeter ruler with an accuracy of 0.5 mm in two perpendicular locations for each sample by three independent observers. Blank disc impregnated with placebo were used as negative control. Each test was performed in triplicate and repeated at least three times.

#### 2.2.3. Biofilm Inactivation Test

Two hundred microliters of MET/DOX gel or placebo were put in the bottom of a sterile eppendorf, and then 2 ml of BHI was added at the top without agitating the gel and incubated at 37°C. At three days, yielding broth containing antibiotic released was collected by a pipette, leaving the underlying layer of gel undisturbed. Two ml of fresh BHI were added back and again incubated at 37°C. The broth samples were collected as described above after 3, 7, 10, 13, and 20 days and used for evaluation of the antibacterial efficacy of MET/DOX gel on biofilm growth. Elution media containing MET/DOX gel or placebo were stored in a refrigerator at 4°C until further use.

For the biofilm assay, *A. actinomycetemcomitans*, *S. sanguinis*, *P. micra*, and *E. corrodens* were cultured in BHI and incubated at 37°C in microaerophilic or anaerobic conditions. Overnight cultures were diluted in fresh BHI or BHI collected as described above (at a ratio of 1 : 100 for culture/BHI) after antibiotic release from the MET/DOX gel. The same procedure was also performed with bacteria biofilm inoculated with placebo. 200 *μ*l of infused BHI thus obtained was inoculated into 96-well flat-bottomed sterile polystyrene microplates (Model 580 BioRad) and incubated for 24 h at 37°C in anaerobic atmosphere. To enhance biofilm formation, the microplates were coated with 20% (vol/vol) human saliva in carbonate buffer (50 mM sodium carbonate, pH 9.5). Saliva samples were collected, after obtaining informed consent, from healthy adult volunteers who were asked not to eat before 2 h prior to collection. The saliva donors were also asked to rinse their mouths gently with water before sampling to decrease bacterial contamination. Samples were subsequently filtered. Five minutes after coating the microplates, saliva was removed and the wells were dried under laminar flow. The biofilm formation was quantified by a modification of a crystal violet assay described by O'Toole and Kolter [56]. Briefly, the biofilm-coated wells of the microtiter plates were washed twice with 200 *µ*l of PBS to remove the planktonic cells and air-dried for 45 min. Each of the wells was then stained with 200 *µ*l of 1% aqueous crystal violet solution for 45 min. The plates were then rinsed with 200 *µ*l of sterile distilled water to remove excess dye and dried to air. The biofilm was quantified by solubilizing crystal violet with a mixture of ethanol and acetone (80 : 20 v/v) and by determining the absorbance of the samples at 570/655 nm (Model 580 BioRad) to determine the amount of biofilm formation. Biofilm growth with placebo was used as a negative control. Each test was performed in triplicate and repeated at least three times. Absorbance values for each bacterial strain at each time point (3, 7, 10, 13, and 20 days) were compared with negative controls with the Student's *t*-test and analysis of variance (ANOVA) for comparison between groups. Significance was considered to be *p* < 0.05.

#### 2.2.4. The Confocal Laser Scanning Microscope (CLSM) Study

The CLSM study was performed only for *S. sanguinis* as a confirmation of the inactivation test of biofilm described above.

Overnight culture of *S. sanguinis*, diluted 1 : 20 in fresh BHI or BHI collected after antibiotic release from the MET/DOX gel, was added to 12-well microplates (Costar; Corning, Inc., NY, USA) containing glass cover slips coated with 20% saliva in carbonate buffer. The microplates containing cell suspensions were incubated for 16 h at 37°C in anaerobic atmosphere. To determine the biofilm formation and the viability of bacteria within the biofilm for the 3, 7, 10, 13, and 20 days, a LIVE/DEAD BacLight staining kit (Molecular Probes, Eugene, Oregon, USA) was used as recommended by the manufacturer [25]. The kit includes two fluorescent nucleic acid stains SYTO9 and propidium iodide. SYTO9 penetrates both viable and nonviable bacteria, while propidium iodide penetrates bacteria with damaged membranes and quenches SYTO9 fluorescence. Dead cells take up propidium iodide and fluoresce red, while viable cells take up fluoresce green. Stained biofilms were examined under a CLSM (Zeiss 510 Meta, Thornwood, NY, USA) using a 20x and 63x oil immersion lens. The excitation and emission wavelengths used for detecting SYTO9 were 488 and 543 nm, respectively. Propidium iodide was excited at 520 nm, and its emission was monitored at 620 nm. The resulting stacks of images were analyzed using the confocal software and subsequently processed using an imaging software (Pro Plus Image Software Media Cybernetics, Rockville, MD, USA). Each sample was analyzed in triplicate and measurements were repeated three times.

## 3. Results

### 3.1. Rheological Measurements

**Figure 2:**
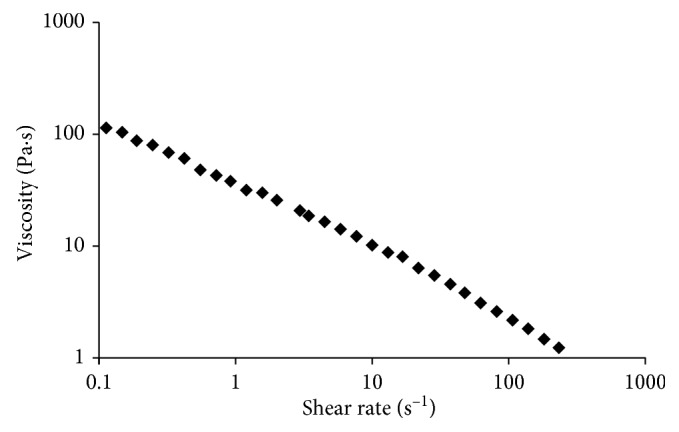
Viscosity vs shear rate for the gel formulation at 25°C.

The viscosity curve of the gel formulation is reported in Figure 2. A pseudoplastic behaviour was evidenced: the viscosity decreased with the increase in the shear rate (flow index value equal to 0.40 ± 0.05). In particular, the viscosity was reduced from 113 ± 2 to 0.8 ± 0.1 Pa·s when the shear rate was increased from 0.1 to 400 s^−1^.

#### 3.1.1. Release Pattern of the Pharmacological Compounds

**Figure 3:**
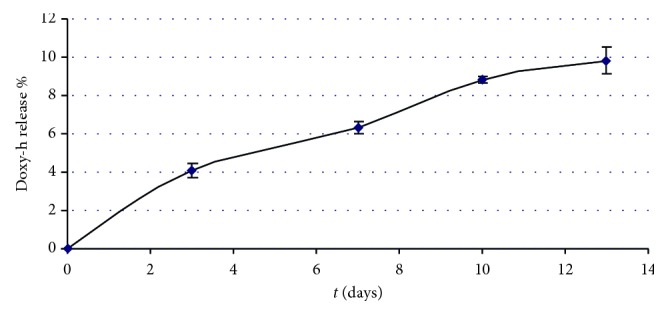
Release of Doxy-h following the change of dialysis fluid up to 13 days (with respect to a negative placebo control).

**Figure 4:**
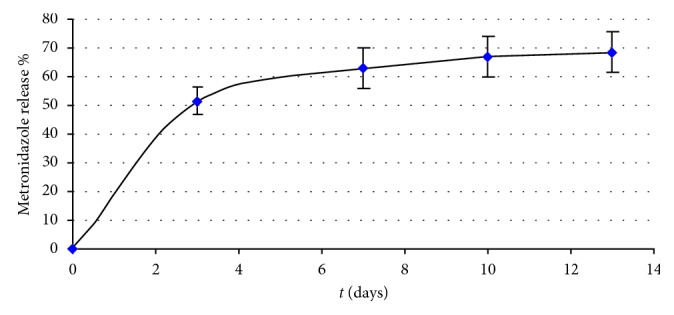
Release of metronidazole following the change of dialysis fluid up to 13 days (with respect to a negative placebo control).

The release found after 13 days was 9.7% (Figure 3) and 67% (Figure 4) of initial dose for Doxy-h and metronidazole, respectively.

The release of Doxy-h at the following intervals was 3 days = 1.2 mg; 7 days = 0.67 mg; 10 days = 0.76 mg; 13 days = 0.29 mg, while the release of metronidazole at the same intervals was 3 days = 30 mg; 7 days = 6.8 mg; 10 days = 2.5 mg; 13 days = 0.9 mg.

#### 3.1.2. Analysis of the Antibacterial Efficacy of the MET/DOX Gel

The inhibitory activity of the Met/Dox experimental gel was tested against *A. actinomycetemcomitans*, *S. sanguinis*, *P. micra*, and *E. corrodens*. The susceptibility to MET/DOX gel for all tested bacterial strains is listed in Table 1. The results at 48 and 72 h were similar with that at 24 h. For all the strains, the mean diameters of bacterial growth inhibition indicated a reasonable agar diffusion of the antibiotics and an inhibitory activity against planktonic bacteria for at least 312 h as compared to negative controls.

**Figure 5:**
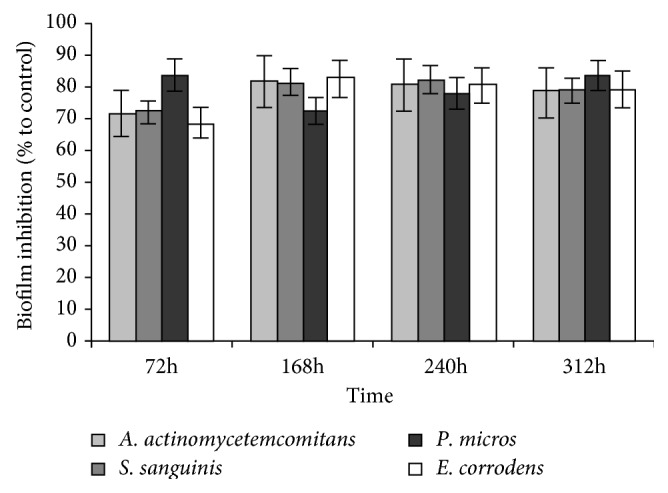
Biofilm inhibition (%) as a function of time, by MET/DOX gel, calculated as a percentage UV absorbance with respect to a negative control, i.e., the absorbance of a biofilm grown in broth without antibiotics released from Met/Dox gel. Bars represent the mean ± SD of triplicate results with separately grown bacteria (*∗P* < 0.05 with respect to the negative placebo control).

Analysis of the inhibition of biofilms formation from MET/DOX gel was studied using *A. actinomycetemcomitans*, *S. sanguinis*, *P. micra*, and *E. corrodens*. The biofilm inhibition after 72 h was 70–80% up to 312 hours (Figure 5) and significantly different from negative controls. MET/DOX gel lost its efficacy after 20 days.

**Figure 6:**
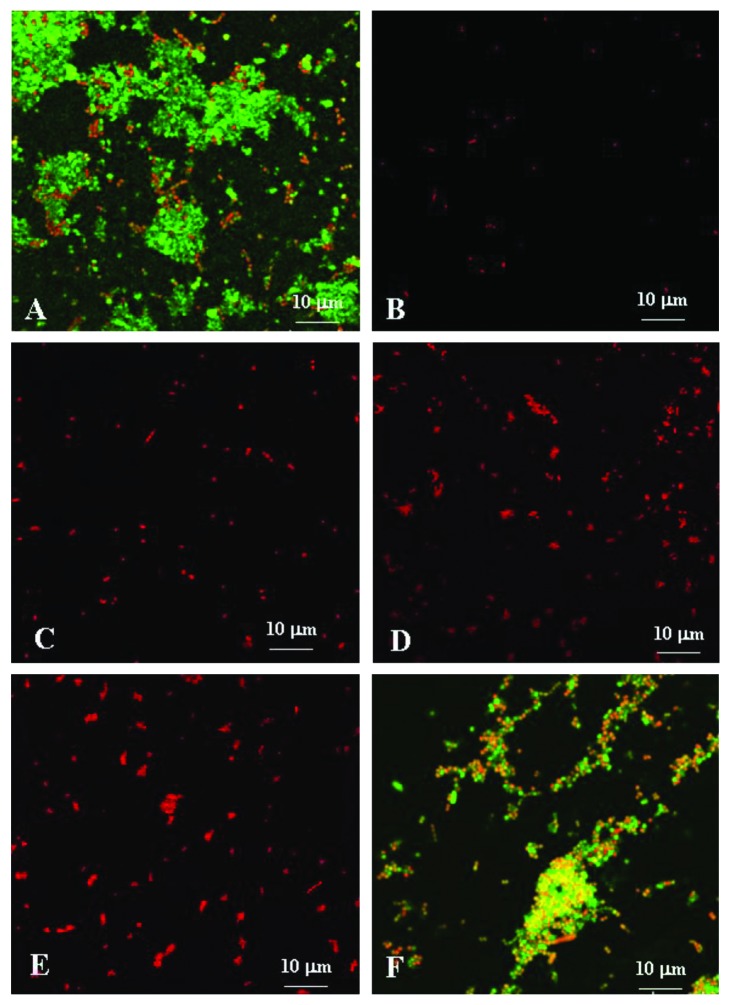
Monitoring *S. sanguinis* biofilm formation on an abiotic surface saliva-coated. Biofilms were stained with the BacLight Live/Dead stain and examined using a Zeiss CLSM510 Meta. (a) Biofilm-negative control (placebo), (b) 3 days, (c) 7 days, (d) 10 days, (e) 13 days, and (f) 20 days. Scale bar = 10 *μ*m (63x magnification).

*Streptococcus sanguinis* biofilms was also examined using a CLSM510. The antimicrobial effects of the media containing metronidazole and doxycycline after elution from MET/DOX gel are reported in Figure 6. The biofilm micrographs obtained in BHI eluted after 3, 7, 10, and 13 days showed a high reduction of biofilm growth. At 20 days, biofilm growth was not inhibited.

## 4. Discussion

This study focused primarily on the development of a gel formulation that was capable of incorporating two different antibiotics (metronidazole and doxycycline) and had slow-releasing abilities. Moreover, the objective was to realize a gel with mucoadhesive properties, exhibiting proper viscosity in order to be syringeable and easily delivered into the pocket.

After testing various concentrations and formulations, we developed a mix of materials that represents an acceptable compromise between adhesion and biocompatibility [57, 58]. The choice of cellulose and polyacrylate as part of the gel material was motivated by their bioadhesive properties [59, 60].

The rheological analyses indicated that the selected formulation exhibited a shear thinning (pseudoplastic) behaviour showing a decreasing viscosity with increasing shear rate (Figure 2). The latter is a desirable property in view of the application: the low viscosity values under high shear rates allow the extrusion through a needle (ease of administration using a syringe), while the high viscosities under low shear rates, such as after injection, prolong the residence in the periodontal pocket [61]. Moreover, an injectable system allows reducing the therapeutic costs compared to devices that need time to be placed and secured at the target site; moreover, it also allows to fill the pocket thus reaching a large portion of the pathogens. Bansal et al. [62] formulated different chitosan-based gels (carried with metronidazole and levofloxacin), with the result of an increasing viscosity at the increase of mucoadhesive properties, so that the best mucoadhesive formulation was not syringeable anymore through a 21-gauge needle. The increase in chitosan content increased also the time of antimicrobial release from the gel, even though it did not go over the rate of some hours.

The gel we formulated had to possess the ability of incorporating and releasing within the pocket the active drugs selected for the antimicrobial activity. Combination of antimicrobial drugs in one delivery system is more effective over single administration as it broadens the spectrum of antimicrobial action; however, it is also difficult to obtain if the molecules are different in terms of sizes and surface properties, as in this case. The two antibiotics used, Doxy-h and metronidazole, were incorporated into the gel at a concentration of 10 mg/ml and 20 mg/ml, respectively. These concentrations were selected on the basis of published data on MIC of 0.25–6.0 *μ*g/ml and 0.1–8 *μ*g/ml, respectively [28, 32]. Gad et al. [63] also developed a solid lipid microparticle encapsulating 5% w/w doxycycline hydrochloride (DH) and 20% w/w metronidazole (MT) for the treatment of periodontal diseases. The in vitro testing of the release showed that up to 80% of the drugs was released between 2 and 8 hours, keeping a very little residual rate up to 80 hours. In the current study, the concentration of drugs incorporated in the gel was greater than the minimum required (approximately 1600 and 2500 times greater for Doxy-h and metronidazole, respectively). This “overfilling” of the gel compensated for the potential loss of gel caused by gingival crevicular fluid and saliva. Moreover, *in vivo*, a partial degradation of the antibiotic due to the temperature and pH of the periodontal pocket is possible to occur. [64, 65]. It was also taken into account the increased MIC when an antibiotic is used *in vivo* against forms of bacteria organized in biofilms [28, 31, 66].

*In vitro* tests showed a controlled release of the two antibiotics from the gel to which they were added, as shown in Figures 3 and 4. Indeed, after 13 days, there was a release in the dialysis medium of 9.7% for Doxy-h (Figure 3) and 67% for metronidazole (Figure 4), with the difference likely due to the molecular weights of the tested compounds {171.2 Da for MET (C_6_H_9_N_3_O_3_; CAS N. 443-48-1) and 512.9 Da for Doxy-h (C_22_H_24_N_2_O_8_·HCl 1/2 (H_2_O) 1/2 (C_2_H_6_O); CAS N. 24390-14-5)} and their structural differences. Nevertheless, the amount of each antibiotic released by the experimental gel was always greater than the MIC reported in the literature for antimicrobial effect, both for bacteria in planktonic and biofilm forms. Indeed, in the first three days, there was a high release of the antibiotics (burst release), 1.2 mg and 30 mg, respectively, for Doxy-h and metronidazole, while a slower release was observed in the following medium changes. Probably, the high release during the first 3 days could be attributed in part to an excess of antibiotics entrapped in the formulation and partly to the antibiotic which is on the outer surface of the gel. In the case of metronidazole, this could be magnified by the size of the molecule known to have an approximately 3-fold lower molecular weight than the Doxy-h. For periodontal application, both effects are of concern in which burst release can be useful to achieve the required MIC, while sustained release is required to maintain drug concentration. Bansal et al. [62] verified an initial burst release in the first six to seven hours followed by slow and sustained release till 48 h. About 70–80% of metronidazole and levofloxacin were released within 6 hours, from the most adhesive and syringeable formulation. Notably in our study, the high initial amount of antibiotic released in the first few days did not compromise the antibacterial efficacy of the gel over longer periods, as the drug release remained sufficiently high to ensure antibacterial activity. Moreover, the burst effect could be advantageous since it is known that the efficiency of antibiotics often depends on the initial drug concentration at the site of infection [67].

In this study, the release in the dialysis medium of Doxy-h, after 13 days, was found to be 0.3 mg, while for metronidazole, the release was higher than 0.9 mg or about 1.5 and 3 times greater than the minimum amount reported in the literature, required to inactivate bacteria responsible for periodontitis [57, 60]. At 13 days, the release was not yet fully exhausted for both antibiotics. Finally, the results of the present study demonstrated that the formulation used herein allowed a convenient release profile of the drugs whilst exhibiting viscosities suitable for injectability, thus representing an attractive system for the MET/DOX delivery in the periodontal or peri-implant pocket.

The bacteria which were used in the study were virulent multispecies community involved in periodontal disease. In particular, *E. corrodens* [68] enhanced the virulence of *S. sanguinis* which is pioneer colonizer [69] and *P. micra* was the most prevalent taxa during oral biofilm formation [70]. By microbiological testings, our results demonstrated the sensitivity of *A. actinomycetemcomitans, S. sanguinis, P. micra,* and *E. corrodens* to the MET/DOX gel at a concentration of 20 mg/ml + 10 mg/ml. Indeed, the growth inhibition occurred as early as 24 hours and was maintained after 48, 72, 96, 120, 144, 168, 240, and 312 hours (13 days), therefore maintaining an *in vitro* efficiency to kill periodontal pathogens.

Other studies showed that the biofilm organization influences the penetration and activity of antibiotics, reducing the effects of the bactericidal action [29–33]. In this study, the quantitative results of inactivation of the biofilm showed a reduction of its formation following the administration of the MET/DOX gel up to 13 days for all strains tested.

Moreover, as viewed by confocal microscopy of biofilms of *S. sanguinis*, there was a bactericidal effect of the MET/DOX gel and a clear disruption of the biofilm for up to 13 days. Only 20 days after the administration of the antibacterial gel, there was a reorganization of the aggregates in the biofilm.

Based on the results of this *in vitro* study, we believe that the release of a combination of two different antibiotics from a controlled-delivery gel was achievable. Although a great effort was made to resemble the in vivo condition of a periodontal environment, the in vitro nature of this study has to be considered as a limit. Because of the complexity and polymicrobial nature of periodontal and peri-implant infections, consisting of both aerobic and anaerobic bacteria, and their complex organization, it is difficult to foresee all the limitations that the clinical use of the gel will arise. However, it seems to be reasonable to plan a clinical study on the base of the efficacy against both planktonic and biofilm species and the prolonged time of effect.

## 5. Conclusions

The gel formulated in the current study obtained positive results for the two phases of experimentation shown above. Indeed, the preliminary test of sensitivity of periodontal pathogens to MET/DOX gel and the test of the extended release were positive. Achievement of minimum inhibitory concentration (MIC) occurred in the first 24 h, and it was held constant for at least 13 days. Inhibition of growth also occurred either by applying the MET/DOX gel before the bacteria or by applying first bacteria and leading to maximum growth, and then subsequently applying the gel, possibly suggesting that the tested MET/DOX gel could be effective both on bacteria that are already present or attempting to colonize the pocket following gel administration. Considering the limitations of this study, future, both *in vitro* and *in vivo*, studies are recommended.

## Figures and Tables

**Table 1:** Zones of microbial inhibition induced by Met/Dox gel (20 mg/ml and 10 mg/ml, respectively) on microaerophilic cultures (*Aggregatibacter actinomycetemcomitans)* or in anaerobic conditions (*Streptococcus sanguinis*, *Parvimonas micra*, and *Eikenella corrodens*) with respect to a negative placebo control = 0 mm for all strains, for all observational intervals.

Inhibition zones (mm)				
Time (hours)	*A. actinomycetemcomitans*	*S. sanguinis*	*P. micra*	*E. corrodens*
24	40 *±* 3	40 *±* 2	40 *±* 3	40 *±* 4
48	38 *±* 2	40 *±* 4	40 *±* 4	37 *±* 3
72	36 *±* 1	35 *±* 2	35 *±* 4	35 *±* 2
96	25 *±* 2	30 *±* 3	32 *±* 2	30 *±* 2
120	25 *±* 1	30 *±* 2	30 *±* 1	27 *±* 3
144	20 *±* 2	27 *±* 1	28 *±* 2	25 *±* 3
168	15 *±* 1	25 *±* 2	25 *±* 1	25 *±* 1
240	14 ± 1	15 ± 3	17 ± 1	15 ± 3
312	12 ± 1	11 ± 2	10 ± 1	10 ± 2

Data are expressed as mean inhibition zone (mm) *±* SD of three replicates.

## Data Availability

The data used to support the findings of this study are included within the article but any additional information is available upon request from the corresponding author.
